# Effect of a computerized decision support system on the treatment approach of stage III or IV pressure injury in patients with spinal cord injury: a feasibility study

**DOI:** 10.1186/s12913-023-09045-y

**Published:** 2023-01-31

**Authors:** Anke Scheel-Sailer, Kamran Koligi, Patricia Lampart, Carina Fähndrich, Hans Peter Gmünder, Stefan Metzger, Dirk Schaefer, Klaus Schmitt, Stefan Stalder, Reto Wettstein, Armin Gemperli

**Affiliations:** 1 Swiss Paraplegic Centre, Nottwil, Switzerland; 2 Department of Health Sciences and Medicine, University of Lucerne, Lucerne, Switzerland; 3 Department of Plastic, Reconstructive, Aesthetic and Hand Surgery, University Hospital Basel, Basel, Switzerland; 4 Swiss Paraplegic Research, Nottwil, Switzerland

**Keywords:** Process Management, Computer Systems, Clinical Decision Support Systems, Basel Decubitus Approach, Interprofessional Collaboration, Pressure Injury, Rehabilitation, Spinal Cord Injury

## Abstract

**Background:**

Stage III and IV pressure injuries (PIs) in patients with spinal cord injury (SCI) require complex interdisciplinary and interprofessional treatment approaches that are difficult to implement. Practical aspects, such as information exchange and coordination, remain challenging. We investigated whether a computerized decision support system (CDSS) could increase treatment adherence and improve clinical outcomes and interprofessional collaboration.

**Method:**

In this feasibility study, a core team developed the initial treatment process and adapted it based on several discussions with clinical experts and information technologists. The CDSS followed the Basel Decubitus Approach and was used in a clinic specializing in SCI. Thirty patients with SCI admitted for stage III/IV PI between July 2016 and May 2017 were randomly allocated to standard or CDSS-supported care. Between-group differences in treatment adherence, complication rates, length of stay, and costs were analyzed using descriptive statistics. The use of the CDSS and potential barriers and facilitators were evaluated through interprofessional focus groups, transcribed verbatim, and thematically analyzed (30 participants).

**Results:**

No differences in SCI characteristics, comorbidities, or PI characteristics (localization: ischium [number (n) = 19 PI, 63%], sacrum [*n* = 10 PI, 33%], recurrent PI [*n* = 21, 70%]) were found between the two groups. Furthermore, no statistically significant differences were observed in treatment adherence, frequency of major (20% vs. 13% between CDSS and control group) and minor (33% vs 27%) complications, and length of stay (98 [±28] vs 81 [±23] days). Healthcare professionals found the CDSS to be helpful for visualizing the treatment process. However, the high workload and difficulties in the information technology processes, such as missing reminders, slow computer performance and data processing, and poor accessibility, hindered the effective implementation of the CDSS.

**Conclusion:**

The implementation of the CDSS to support the treatment of stage III/IV PI in patients with SCI was feasible and included definitions of milestones, interventions, and outcomes. However, to assess the impact of the CDSS, a longer observation period is required. Further, the technical difficulties must be addressed, and solid integration of the CDSS into the clinical information system is necessary.

**Trial Registration:**

This quality improvement project received a declaration of no objection from the Ethics Committee of Northwest and Central Switzerland (EKNZ UBE-16/003), and ethical approval was received for the focus groups (EKNZ Req-2017-00860).

**Supplementary Information:**

The online version contains supplementary material available at 10.1186/s12913-023-09045-y.

## Introduction

Process management is an innovative methodological step in healthcare [1–6]. It includes a series of defined diagnostic and therapeutic interventions for treating a problem, achieving a goal, shaping and structuring different clinical settings, and improving guideline adherence. However, the implementation of process management in many clinical settings is challenging due to a lack of awareness of all process elements, a lack of knowledge translation, and the complexity of treatment [2]. Hence, computerized decision support systems (CDSSs) were developed to improve guideline adherence by leading health-care professionals (HCPs) through these processes, reminding them of relevant steps, and managing them in their specific duties [3]. CDSSs optimize adherence to treatment processes, reduce teams’ workloads, and consequently reduce total treatment costs [1, 3]. Most importantly, standard treatment processes may be reevaluated and continuously improved by using data from daily clinical management. To date, CDSSs have mainly been evaluated for the management of acute clinical situations. They have been shown to lead to reduced complication rates [7], increased employee satisfaction [8, 9], better communication during visits, and a better overview of information for all involved HCPs [10, 11]. While CDSSs are tools for quality improvement and influence quality indicators [11, 12], contradictory results have been obtained from process management and CDSS implementation in clinical contexts, regardless of whether the CDSS led to improved guideline adherence or to a reduction in complication rates [5, 7].

According to the European Pressure Ulcer Advisory Panel (EPUAP), the treatment of stage III and IV pressure injuries (PIs) in patients with spinal cord injury (SCI) consists of acute care and rehabilitation provided by various HCPs. The interdisciplinary team includes different physicians, such as plastic surgeons, paraplegiologists, internal medicine physicians, and infectiologists as well as HCPs from other professions, such as nurses, physio- and occupational therapists, psychologists, and social workers [13]. Due to the complex coordination needs in interdisciplinary treatment, this treatment could benefit from process management and CDSS-supported therapy to achieve clearly structured procedures. PIs are among the most frequent and cost-intensive complications in people with SCI [14, 15]. Often aggravated by early and late postsurgical complications [16–20], PIs can lead to long hospital stays and reduced quality of life [21, 22]. Internationally, it is accepted that stage III/IV PIs require flap reconstruction [17, 19, 22], postsurgical immobilization, and antibiotic therapy [23]. Because complications during early postsurgical treatment occur more often in patients with a high-risk profile, individual risk analyses are recommended, such as malnutrition screening, as well as the diagnosis and treatment of comorbidities [19, 24–26].

The Basel Decubitus Approach is a set of principles for the treatment of PIs that has been developed over the past few decades. It includes different therapeutic elements, such as pressure relief; debridement; treatment of risk factors, such as anemia, renal failure, uncontrolled diabetes, and spasticity; flap surgery; prevention of early postsurgical complications and PI recurrence; transfer training; and re-evaluation of assistive devices [21, 25–29]. Due to the complexity of this treatment approach, its implementation and adherence in the clinical setting have been difficult [18, 21, 26, 28, 30]. We hypothesized that a CDSS would support process management, thus increasing adherence to the Basel Decubitus Approach and improving the quality of treatment for patients with SCI and stage III and IV PIs by reducing complications, shortening the length of stay, and improving secondary prevention strategies [31].

This feasibility study aimed to describe the effect of a CDSS on adherence to the Basel Decubitus Approach by comparing complication rates, length of stay, total-, intervention-, and occupation-specific costs, and the performance of examinations and interventions between standard and CDSS-supported care. Additionally, this study explored HCPs’ perspectives on the use of this CDSS.

## Methods

### Design

We conducted a feasibility study with routinely collected data for our quality improvement project and a qualitative focus group study. For reporting, we used the CONsolidated Standards of Reporting Trials (CONSORT) 2010 extension pilot or feasibility trial checklist [32, 33] (Appendix 1).

### Setting

This study was conducted at a Swiss acute and rehabilitation clinic that specializes in SCI. This clinic integrates a plastic surgery team from a nearby university hospital and is specialized in the treatment of PI in patients with SCI using the Basel Decubitus Approach. The Basel Decubitus Approach [26–28, 34] is based on six main principles: pressure relief (1); debridement (2); treatment of risk factors and comorbidities, (3) such as anemia, renal failure, uncontrolled diabetes, and spasticity; wound conditioning (4), flap surgery (5); and prevention of early postsurgical complications and PI recurrence (6). Some interventions, such as nutritional and psychological counseling, have redefined and integrated into the Basel Decubitus Approach [25, 29, 30].

The treatment approach for PI includes paraplegiology, plastic surgery, infectious disease management, specialized acute and rehabilitation care, physical therapy, occupational therapy, nutritionist support, and psychotherapy [21, 24, 27–30].

### Participants

We included adult patients with SCI or similar syndromes (e.g., multiple sclerosis) presenting with a stage III/IV PI according to the EPUAP classification over the ischium, sacrum, or trochanter who were admitted for inpatient treatment, including plastic surgery. Patients with a malignant disease, a local skin disease (e.g., fungal infection), or who denied use of their data were excluded.

All patients meeting the inclusion criteria between July 2016 and May 2017 were randomly assigned by the admission case management team to standard care or to treatment by specially trained teams using the CDSS. Three out of six teams in the clinic in different wards were trained to use the CDSS. The admission case management team, whose members were not informed about the CDSS use, simply assigned patients to “free” beds and distributed the workload equally among the nursing wards. The admission case management team was blinded to the assignment of patients to wards, and the research team was not involved in the ward assignment process.

### Intervention

#### Development of the CDSS

The objective of the CDSS was to illustrate interprofessional management according to the Basel Decubitus Approach and to guide the HCPs through the complex treatment intervention. The development of the CDSS involved four steps.

First, during the preparation phase from 2015 to 2016, the core team (a specialized physician [AS-S], a wound care nurse [KGL], a quality management expert [KS], and an information technology (IT) specialist [SS]) defined the use case. A “use case” is the starting point in the IT logic for creating a business process modeling notation (BPMN). It includes a list of actions or event steps in this specific process. The core team defined the milestones during the treatment process. For example, after the flap surgery the patient was immobilized on an airflow mattress. If there were no complications, the suture material was removed 21 days after the surgery. In case of delayed wound healing, wound dehiscence, or other complications, such as hematoma or infection, the timing of the suture removal was determined individually by the plastic surgeon. Influential dependencies between each step were described and visualized according to the Basel Decubitus Approach. The core team determined that the CDSS should guide the HCPs through the treatment process and make the process visible. The CDSS informed the physicians of the list of required steps during the treatment process (prescription of various assessments, examinations, and other therapies). The CDSS guided the interprofessional team through the complex treatment process with relevant milestones, which were integrated into their clinical management. The IT process and milestones were adapted if required based on clinically indicated individual changes in treatment.

**Fig. 1:**
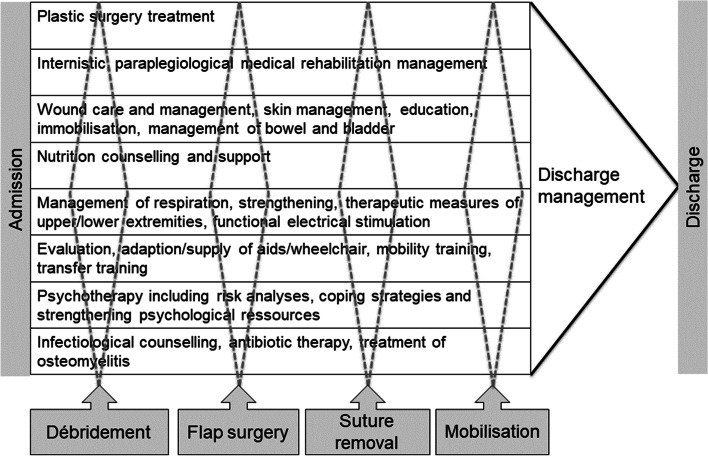
Treatment principles and milestones in the “ Basel Decubitus Approach”. Authors own work, copyright @anke.scheel-sailer

Second, we defined the measurement of baseline assessments, milestones, timelines, and outcomes according to the Basel Decubitus Approach (Table 1). The process combined the following milestones: admission, debridement and bone biopsy, flap surgery, suture removal, mobilization, and discharge (Fig. 1). Nine general therapeutic interventions, seven assessments, five consultations, and all professional-orientated responsibilities were defined (Appendix Table 1). In the presence of osteomyelitis, an infectious disease specialist was also involved. The core team met monthly for coordination.

**Table 1:** The “Basel Decubitus Approach” structured to focus, profession and content

Focus	Profession	Content
Classification of PI	Paraplegiologist Plastic Surgeon Nurse	Classification of PI according to definition of the EPUAP and NPUAP (reference). Documentation following international recommendations (reference) including photos
Pressure relief	Paraplegiologist Plastic Surgeon Nurse	Immediate immobilization after admission for inpatient treatment either on airflow mattresses or with supine ventral positioning, if possible.
Local dressing	Paraplegiologist Plastic Surgeon Nurse	Application of dressing according to the TIME concept. Removal of necroses, treatment of infection (local disinfection and/ or surgical debridement) and negative pressure therapy or three times per day wet dressings.
Diagnostic and treatment of risk factors	Paraplegiologist	Standard blood analyses: inflammation, anaemia, electrolytes, kidney and liver function and nutrition profile
	Dietitician	The SNST (Spinal Cord Nutrition Tool) is administered to detect nutrition and nutrition. Counselling is added to address the special needs during the treatment of the PI and respect the changed protein and caloric requirements
	Paraplegiologist	Screening of neurological changes and examination of the international standard for neurological classification in SCI.
	Paraplegiologist Nurse Physical therapy	Examination of pulmonary function. Breathing therapy and exercise.
	Paraplegiologist Physical therapy Occupational therapy	Examination of the lower extremities (range of motion) and of the spine (scoliosis?) and evaluation of the seating position.
	Psychologist	Screening of psychological risk factors and integrated psychotherapy by individual indication.
Flap surgery	Plastic surgeon	Closure of PI with fasciocutaneous tissue if possible: os ischium – posterior thigh flap, os sacrum/ coccygis – gluteal rotation flap, os trochanter – lateral thigh flap/tensor fasciae latae flap.
Diagnosis and therapy of osteomyeliltis	Plastic surgeon Paraplegiologist Infectiologist	Bone biopsies during the surgical debridement or flap surgery. Bacterial diagnostic and empirical and targeted infection treatment with antibiotics about 6 weeks if osteomyelitis.
Postsurgical immobilisation	Plastic surgeon Paraplegiologist	According to the diagnosis of osteomyelitis and recurrence the postsurgical bed rest is 4 or 6 weeks.
Prevention of secondary complication or recurrence	Interdisciplinary and interprofessional team	Individual risk analyses Evaluation of seating position and cushion Evaluation of transfer techniques and strengthening of the upper extremity

Third, consensus conferences were held with experts from all disciplines involved (physician specialists for paraplegiology; a plastic surgeon; an infectiologist; nurses; physio-, occupational and nutritional therapists; and a psychologist) to review the first draft and describe the use case based on key principles of the Basel Decubitus Approach. Several adaptations were necessary, including feedback systems, dependencies between different sub-processes, the need and controlling for assessments, consultations, and visibility.

Fourth, IT specialists were responsible for converting the use case into a BPMN. The CDSS was pilot tested in three cases with the responsible interprofessional team during the pre-test period. The core team participated in weekly meetings in which they demonstrated and documented the treatment procedures. After the pilot testing, dependencies between suture removal and changes in mattresses were no longer automatically connected due to frequent individual changes. In the CDSS, three key aspects of the process were visible: treatment elements, consultations, and milestones (Appendix Fig. 2). HCPs could see an overview of the entire process: they could click on the CDSS picture in a field showing the different treatment elements and were led to prescribe different specialist consultations and therapies. As soon as these consultations were prescribed, the color of the field in the process picture turned green. Once all consultations and therapies were prescribed, the color in the overview level also changed to green. The development of the CDSS required about 300 hours of interprofessional engagement and about 10,000 hours of IT work.

#### Implementation of the CDSS

When the development phase was completed, the involved interprofessional teams were informed, and a specific training program based on a structured manual was organized in June 2016. Specifically, the core team offered three interprofessional team information events, which lasted about 60 minutes each, with representatives of all professions (junior physicians, senior consultants, nurses, physio- and occupational therapists, social workers, and psychologists). In addition, individual support was offered on demand.

### Patient-related data collection

The following data were gathered manually from the hospital records: patient, SCI, and PI characteristics; osteomyelitis; examinations; and interventions as they were defined as milestones and outcome parameters (minor and major complications, length of stay). The following information on examinations, interventions, and milestones was collected: SCI neurological impairment based on the International Standard for Neurological Classification in SCI (ISNCSCI), electrocardiogram, pulse oximetry, spirometry, nutrition laboratory, and range of motion. To monitor the treatment process and adherence to the Basel Decubitus Approach, we collected data on the prescription of plastic surgery consultation; physical, occupational, nutritional, psychological, music, and/or art therapy; the beginning of physical, occupational, nutritional, psychological, music, and/or art therapy; the performance of flap surgery; leg movement; and first mobilization in a wheelchair. The workload of physicians per patient was documented in five-minute steps for different tasks (direct patient contact, interprofessional exchange, documentation). Complications were divided into four categories: no complications, minor complications (prolonged bedrest), and major complications (re-surgery) directly related to the surgical intervention and other independent complications, such as pneumonia or urinary tract infection.

### Cost analysis/calculation

The length of hospital stay was used to calculate the total costs, as the reimbursement included day taxes. Individual real hospital costs were collected as patient cost calculations performed by the finance department. Specific treatment costs were analyzed in terms of the costs of plastic surgical care, therapies, nursing, laboratory medical examinations, medication, and physicians’ time.

### Statistical analyses

Categorical variables were presented with absolute and relative frequencies separately for the CDSS group, the control group, and all patients. Continuous variables were presented with mean or median lower and upper quartiles for each group. The continuous variables were divided into respective categories, ranging from normal to slightly deviant to strongly deviant values: body mass index (BMI) (< 17, 17–24, > 24 m/kg^2^) [35], hemoglobin (< 80, 80–120, < 120 g/l), vitamin D (< 30, 30–75, > 75), and glomerular filtration rates (GFR) (< 30, 30–90, > 90 mm/l). A time-to-event analysis was carried out to assess the mean time between admission and milestones or interventions of the Basel Decubitus Approach between the CDSS and control groups. *P*-values for the group comparisons were computed using chi-square tests and Fisher’s exact test for categorical variables. We used the unpaired two-sample t-test for continuous variables. The alpha level for statistical significance was set at 0.05. Data preparation and statistical analysis were conducted using Stata SE 15 (Stata for Windows, College Station, TX, USA).

### Evaluation of the user perspective

In November and December 2017, we conducted four profession-specific focus groups (junior physicians, nurses, physio- and occupational therapists, and senior consultants) with five to eight participants, which lasted approximately 60 minutes each (Appendix Table 3). All participants had experience with the CDSS during the pilot testing. The semi-structured interview guide covered questions concerning experiences (“What experience have you had using the process management system?”), possibilities (“What tasks should such a system perform?”), and challenges (“What negative impact could such a system have on your activities?”) related to the newly implemented CDSS. These focus groups were audio recorded, transcribed verbatim, anonymized, and thematically analyzed [35]. We encoded the data using ATLAS.ti software (ATLAS.ti Scientific Software Development GmbH, Berlin, Germany). KK, PL, and AS-S grouped the obtained codes into subthemes and identified recurring themes through an iterative process.

## Results

### Patient and disease characteristics

Each group had 15 patients, and no dropouts occurred during the observation period. The patient characteristics are presented in Table 2. Twenty-two participants were male (73%), and the mean age was 56 years (interquartile range: 42–70 years). The two groups were equally distributed in terms of demographic factors. Nineteen patients had a complete SCI (63%), and 11 had a cervical SCI (37%). Comorbidities, such as diabetes, hypertonia, and renal failure, were equally frequent between the groups. Time since injury (means of 18 and 23 years in the CDSS and control groups, respectively) and BMI (means of 20.8 kg/m^2^ and 24.6 kg/m^2^ in the CDSS and control groups, respectively) were similar between the groups (Table 2).

**Table 2:** Patient characteristics overall, in the process management group and in the standard care group (gender, etiology, completeness and level of lesion, comorbidities, years since injury, body mass index)

Parameter	Category	Total (***N*** = 30)	CDSS Group (***N*** = 15)	Control Group (***N*** = 15)	***P***-value**
*Categorical Parameters – n (%)*					
Sex	Male	22 (73)	10 (67)	12 (80)	0.409
	Female	8 (27)	5 (33)	3 (20)	
Etiology of SCI	Transport Activity	8 (267)	4 (27)	4 (27)	0.844
	Sports/Leisure Activity	4 (13)	3 (20)	1 (7)	
	Fall	4 (13)	2 (13.)	2 (13)	
	Other Accident Cause	4 (13)	2 (13)	2 (13)	
	Other or Unknown^a^	10 (33)	4 (27)	6 (40)	
Completeness of Lesion	Complete	19 (63)	8 (53)	11 (73)	0.256
	Incomplete	11 (37)	7 (47)	4 (27)	
Lesion Level	C1-C4	5 (17)	3 (20)	2 (13)	0.512
	C5-C8	6 (20)	4 (27)	2 (13)	
	T1-S5	19 (63)	8 (53)	11 (73)	
*Comorbidities – n (%)*					
Diabetes Mellitus		6 (20)	1 (7)	5 (33)	0.068
Coronary Heart Diseases		5 (17)	2 (13)	3 (20)	0.624
Arterial hypertension		13 (43)	6 (40)	7 (47)	0.713
Vitamin D Deficiency		24 (80)	12 (80)	12 (80)	1.000
Anemia		23 (77)	12 (80)	11 (73)	0.666
**Arterial occlusive disease**		6 (20)	2 (13)	4 (27)	0.361
Arteriosclerosis		5 (17)	2 (13)	3 (20)	0.624
Psychiatric Diagnosis		7 (23)	5 (33)	2 (13)	0.195
*Continuous Parameters – Median (Q1;Q3)*					
Age (years)		56.2 (42.0;69.7)	53.7 (36.7;74.4)	56.5 (51.7;69.7)	0.419
Years since SCI		19.4 (9.2;33.1)	17.8 (9.1;25.0)	22.9 (10.7;34.7)	0.395
BMI (kg/m^2^)		23.1 (19.8;26.0)	20.8 (16.6;25.8)	24.6 (22.7;27.3)	0.065

^a^Includes multiple sclerosis, inflammatory and iatrogenic caused by surgical intervention

** Chi-square tests and Fisher’s exact test for categorical and unpaired two-sample t-test for continuous variables

### Clinical outcome: Complication rates

PI characteristics, including the localization (*n* = 19 PI over the ischium [63%]), recurrence (21 patients, 70%), treatment with a rotation flap (12 patients, 40%), and treatment with a posterior thigh flap (15 patients, 50%), did not statistically differ between the groups (Table 3). The five major (16%) and nine minor (39%) complications were distributed similarly between the groups. Likewise, the length of stay (90 days on average) as well as the overall and detailed intervention costs did not differ significantly between the groups (Appendix Table 4).

**Table 3:** Characteristics of PI (number of PIs, recurrent or primary PI, grade, localization), intervention (type of flap surgery) and outcome parameters (flap and general complications)

Parameter	Category	Total (***N*** = 30) n (%)	CDSS Group (***N*** = 15) n (%)	Control Group (***N*** = 15) n (%)	***P***-value*
Total Number of	1	19 (63)	8 (53)	11 (73)	0.321
Pressure Injuries	2	6 (20)	3 (20)	3 (20)	
	3+	5 (17)	4 (27)	1 (7)	
Number of recurrence of Pressure Injuries		21 (70)	10 (67)	11 (73)	0.690
Grade of Pressure	III	5 (17)	3 (20)	2 (13)	0.624
Injury	IV	25 (83)	12 (80)	13 (87)	
Osteomyelitis		22 (730)	11 (73)	11 (73)	
Localization	Ischium	19 (63)	10 (67)	9 (60)	0.484
	Sacrum	10 (33)	4 (27)	6 (40)	
	Trochanter	1 (3)	1 (67)	0	
Type of Flap	Gluteal Fasciocutaneous Rotational Flap (Sacrum)	12 (40)	5 (33)	7 (47)	0.693
	Posterior Thigh Flap (Ischium)	15 (50)	8 (53)	7 (47)	
	Other	3 (10)	2 (13)	1 (7)	
Complications	None	16 (53)	7 (47)	9 (60)	0.755
	Minor (Prolonged Bedrest)	9 (30)	5 (33)	4 (27)	
	Major (Resurgery)	5 (17)	3 (20)	2 (13)	
Type of Additional	None	24 (80)	13 (87)	11 (73)	0.558
Complications	Pneumonia	4 (13)	1 (7)	3 (20)	
	Urinary Tract Infection	2 (67)	1 (7)	1 (7)	
Length of Stay (days)	Mean (SD)	90 (26)	98 (28)	81 (23)	0.124
	Median (Q1; Q3)	80 (71; 117)	89 (76; 120)	78 (71; 86)	
	Min; Max	54; 147	55; 147	54; 140	

*Abbreviation*: *CDSS* Computerized Decision Support System

* Chi-square tests and Fisher’s exact test for categorical and unpaired two-sample t-test for continuous variables

### Treatment approach adherence

A trend toward more prescriptions of therapies and examinations was observed under the CDSS (Table 4). For example, physical and occupational therapy was prescribed to 15 patients (100%) in the CDSS group and 13 patients (87%) in the usual care group; however, therapy took place in both groups equally (Table 3). Psychology was prescribed in 12 patients (80%) in the CDSS group compared to nine patients (60%) in the usual care group. The beginning of psychological treatment differed less between the CDSS group (mean 13.4 days; confidence interval (CI) 8.3–18.4) and the usual care group (mean 27.6 days; CI 6.1–49.0). Pulse oximetry, as an example of an examination, was conducted in seven patients (47%) in the CDSS group compared to five (33%) in the usual care group. All examinations were performed in accordance with the Basel Decubitus Approach. There was no statistically significant difference in the proportion of patients in the CDSS group vs. the control group regarding the prescription of additional therapies, such as psychological or nutritional therapy (Table 4).

**Table 4:** Absolute and relative frequencies of ordered interventions and time from admission to intervention by intervention and control group

	Frequencies		Ratio	Odds Ratio		Mean Time-to-Event in Days				Mean Difference	
Interventions	CDSS Group ***N*** = 15 n (%)	Control Group ***N*** = 15 n (%)	CDSS1 CDSS0								
				OR	CI (95%)	CDSS Group		Control Group		μ1-μ0	CI (95%)
						μ1	CI (95%)	μ0	CI (95%)		
Prescription Physical therapy	**15 (100)**	13 (87)	1.15			0.87	−0.33-2.07	0.08	− 0.55-0.70	− 0.79	−2.14-0.57
Begin Physical therapy	**15 (100)**	**15 (100)**	1.00			1.53	0.53–2.53	1.00	0.34–1.66	−0.53	−1.68-0.61
Prescription Occupational Therapy	**15 (100)**	13 (87)	1.15			0.87	−0.33- 2.07	0.08	−0.55-0.70	− 0.79	− 2.14-0.57
Begin Occupational Therapy	**15 (100)**	**15 (100)**	1.00			1.47	0.34–2.59	3.20	−0.67-7.06	1.73	−2.11-5.57
Prescription Psychology	12 (80)	9 (60)	1.44	2.67	0.41–20.45	**10.25**	0.16–20.37	**20.67**	−3.16-44.50	**10.42**	−11.2-32.07
Begin Psychology	11 (73)	9 (60)	1.33	1.83	0.31–11.63	**13.36**	8.30–18.43	**27.56**	6.14–48.97	**14.19**	−4.13-32.52
Prescription Music & Art therapy	8 (53)	4 (27)	2.00	**3.14**	0.55–19.56	26.50	4.18–48.82	19.25	−20.51-59.01	−7.25	− 42.99-28.49
Begin Music & Art therapy	7 (47)	4 (27)	1.75	2.41	0.416–15.02	35.71	15.17–56.26	29.00	−15.46-73.46	−6.71	−41.13-27.70
Prescription Nutritional Counseling	**15 (100)**	13 (87)	1.15			0.53	−0.25-1.31	1.08	− 0.93-3.09	0.54	−1.39-2.48
Begin Nutritional Counseling	14 (93)	12 (80)	1.17	**3.50**	0.23–196.59	5.86	3.33–8.39	5.83	3.64–8.03	−0.02	−3.26-3.21
Nutrition Laboratory	**15 (100)**	**15 (100)**	1.00			0.87	−0.55-2.28	0.07	−0.08-0.21	− 0.80	−2.16-0.56
Prescription Pulse Oximetry	8 (53)	6 (40)	1.33	1.71	0.32–9.25	18.00	−3.29-39.30	19.00	−1.89-39.89	1.00	−26.44-28.44
Examination Pulse Oximetry	7 (47)	5 (33)	1.40	1.75	0.32–9.92	24.29	−3.75-52.32	27.60	1.95–53.25	3.31	−31.75-38.38
Prescription Spirometry	6 (40)	5 (33)	1.20	**3.33**	0.11–235.24	**4.33**	−0.66-9.33	**35.40**	−15.71-86.51	**31.07**	−6.84-68.97
Examination Spirometry	4 (27)	2 (13)	2.00	2.36	0.27–30.02	**8.00**	−3.55-19.55	**38.00**	− 381.30-457.30	**30.00**	−28.11-88.11
ISNCSCI	5 (33)	7 (47)	0.71	0.57	0.10–3.13	**35.40**	−15.41-86.21	**41.00**	21.29–60.71	**5.60**	−34.45-45.65
Joint status	**15 (100)**	14 (93)	1.07			**12.13**	1.94–22.33	**24.00**	3.61–44.39	**11.87**	−9.38-33.11
Prescription ECG	9 (60)	10 (67)	0.90	0.75	0.13–4.23	**2.00**	−0.34-4.34	**9.70**	−10.32-29.72	**7.70**	−12.14-27.54
Examination ECG	9 (60)	9 (60)	1.00	1.00	0.18–5.46	**3.56**	0.97–6.14	**11.00**	−11.78-33.78	**7.44**	−13.63-28.52
Prescription Plastic Surgery	12 (80)	10 (67)	1.20	2.00	0.29–15.84	1.58	−0.05-3.22	0.00	− 0.34-0.34	−1.58	−3.31-0.15
Flap Surgery	**15 (100)**	**15 (100)**	1.00			22.27	15.91–28.63	21.53	11.60–31.47	−0.73	−12.00-10.53
Ordinance 4 / 6 Week Schedule	**15 (100)**	14 (93%)	1.07			22.27	15.91–28.63	22.00	11.31–32.69	−0.27	−11.92-11.39
Moving Legs	14 (93)	**15 (100)**	0.93			63.79	52.40–75.18	54.53	43.88–65.19	−9.25	−24.10-5.60
First Mobilization in Wheelchair	**15 (100)**	**15 (100)**	1.00			74.20	61.80–86.60	62.07	51.44–72.69	−12.13	−27.73-3.47
Discharge						98.07	−0.33-2.07	81.33	− 0.55-0.70	−16.73	−38.95-2.49

*Abbreviation*: *CDSS* Computerized Decision Support System, *ISNCSCI* International Standard for Neurological Classification Spinal Cord Injury, *ECG* Electrocardiogram, *μ0* Mean of control group, *μ1* Mean of CDSS group

### Feedback from healthcare professionals and evaluation of user perspectives

The focus groups included 30 HCPs (26 female, 87%), consisting of eight nurses, six physiotherapists, three occupational therapists, seven junior physicians, and six senior consultants with 405 collective years of work experience and, more specifically, 231 collective years of work experience in this clinic (Appendix Table 3). Participants in all groups mentioned topics concerning IT process requirements and workload, clinical relevance, and the meaningfulness of the CDSS (Table 5). A benevolent willingness to use the CDSS became apparent (Questions [Qs] 1, 2, and 3). Participants acknowledged that the CDSS supported the use of guideline recommendations (Q4). Moreover, the HCPs expressed their awareness of directing the system, that is, that they could use it and adapt it individually if needed (Q5). While different HCPs underlined the opportunity to obtain a good interprofessional overview using the CDSS (Q6), others criticized what they regarded as a poor overview (Q7). HCPs often felt that the level of detail was too high (Qs 8 and 9).

**Table 5:** Quotes about experiences using the CDSS (advantages and challenges)

1.	Overall, I think it is useful (resident physician)
2.	For me the outline of the process for the Decubitus was valuable. (senior consultant)
3.	The overview as a sort of checklist was good (junior physician)
4.	I thought it was like guidelines. You were reminded whether it is time to change something – I asked myself the questions: is it correct, can we do this now? It is like following a standard process (nurse expert)
5.	You don’t necessarily have to do it then; you can also reset or change it. (nurse expert)
6.	When the tool was presented, I was very optimistic. I had the feeling this could be very interesting, especially knowing what phase the patient is in or what the physio is doing and what ergotherapy is he receiving? (nurse expert)
7.	It is extremely confusing. I continue to read the processes from Medfolio. The PMC is very unclear. (junior physician)
8	On the one hand a ton of potential, also for handovers between different steps, on the other hand a huge risk to get lost in detail. (therapy expert)
9.	I think this may be the biggest issue, it is too detailed (senior consultant)
10.	Filling in the required information in the system took up more time than we saved through the process, this is a complete no-go. (senior consultant)
11.	For me the accessibility and speed of our IT system are completely useless. (senior consultant)
12.	In the end things did not work as we planned. For example, logging into the system did not always work. (nurse expert)
13.	You need to look for the patient, then you have to click management, then you need to go somewhere else [in the system] … somehow there is no overview (resident physician)
14.	You always have to take the extra step to open it. It does not automatically open to remind you that something is still red. You actively have to open it (junior physician)
15.	Doing this twice, or rather duplication is definitely a big topic for our IT-system. It would be great if we could get rid of this, e.g. by things populating automatically once the information is filled in in one location. (nurse expert)
16.	And then, it does not autocomplete, you need to activate and confirm it. It does not simply happen once you entered the information. (nurse expert)
17.	The main issue was that it is various systems in which we work. (senior consultant)
18.	Since we work in so many different programs it eats up your time to work according to this process. (junior physician)
19.	I want to work scientifically. Instead of the system getting the required information itself, you need to go from one section to another to get the data yourself. In the end you feel like Sisiphus. (senior consultant)
20.	One did not really look at the system. It was simply used for the others, so they could draw conclusions. (nurse expert)
21.	I would claim you have also witnessed it when you substituted me. The regular employee did not do anything with the system. It was only the team-leader when he visited the patient, not the regular employee who is actually affected, who used the system. The regular employee had no clue. (therapy expert)
22.	I have the impression that in this process we want to collect data, as you said, and want to analyze and use it for research. (junior physician)
23.	After a while we noticed that other departments or also we forgot to enter the information. Things are just done so automatically. One is caught up in old behavior patterns. (nurse expert)
24.	It just takes longer but the quality is not better. (therapy expert)
25.	It was simply completing a task. It did not help us in our clinical day to day work, it was simply an externally imposed duty. (therapy expert)

*Abb.*: *PMC* Patient management cockpit (the CDSS = computerized decision support system

All HCPs complained about the high time effort required for the active maintenance of the CDSS (Q10), the slowness of the IT system (Q11), and the instability of the IT system (Q12). The whole process was not automatically initiated or self-explanatory (Q13). Automatic reminders were not yet technically feasible (Q14). An immense problem appeared concerning the duplicity in the documentation. Since different IT systems were used in parallel, information had to be documented in different places (Qs 15, 16, 17, and 18). However, collected data could not be automatically displayed in different places and used in different processes (Q19).

HCPs could not develop a clinical management routine due to the rare use of CDSS with only a small number of patients per ward (Q20). Therefore, the CDSS did not noticeably support information exchange in staff shift changes (Q21). For some users, the purpose of the CDSS was perceived as limited to collecting data for research, leading to less motivation and more resistance to its use (Qs 19 and 22). Finally, HCPs were accustomed to paper-based process management (Q23) and did not experience improved quality when using the CDSS (Q24), only a compulsory task (Q25).

The feasibility study stopped in June 2017 because the management board finished the use of this specific IT system due to high costs, an inability to manage the interfaces, a lack of applicability to all IT users in the clinic, and the inability of the IT company that developed the system to fulfill all requirements. The management board finally decided to use another IT system in the whole clinic and to stop the use of this specific IT system that had explicitly developed the CDSS. Therefore, the developed CDSS could no longer be used for clinical management.

## Discussion

To the best of our knowledge, this is the first feasibility study on the use of a CDSS in the treatment of PI in patients with SCI. The prescriptions and therapies were carried out almost equally in both groups. Nevertheless, in the CDSS group, the prescriptions and therapies generally started earlier than in the control group. The use of the CDSS did not significantly improve clinical and economic outcomes during this observation period. All HCPs showed interest in the CDSS and a willingness to integrate it into their clinical routine. HCPs endorsed the CDSS as a way to improve the quality of patient care by integrating guideline recommendations, allowing individual adaptations, and supporting interdisciplinary and interprofessional treatment. However, technical difficulties and time-consuming tasks characterized the use of the CDSS, which hindered its use as an extra IT system.

Different attitudes and principles between the clinical and the IT management group became apparent [3] in the monthly discussions of the core team. The importance of mutual understandings of the perspectives of the interprofessional clinical team and the IT team during the development process was evident during the consensus conferences [3]. As reported in the literature, clinical decision-making is characterized by its complexity and flexibility in adapting to individual patients’ situations. This appeared to contradict the IT logic of clear and linear causalities and predictable dependencies [3]. To overcome the challenge of these different professional perspectives and work methods, it was necessary to elaborate the benefits of a consensus based clinical management and to clarify the most relevant aspects of the treatment approach and interprofessional coordination. The interprofessional dependencies between activities of different professions had to be clearly defined to transform the collaborative processes into a clear IT logic. This study demonstrated that use case development was feasible in this interdisciplinary and interprofessional treatment process [3] when detailed information about the interprofessional and interdisciplinary interventions, milestones, and treatment elements could be elaborated [17, 19]. The relevant milestones and treatment elements in the Basel Decubitus Approach include debridement, surgical procedures, diagnosis and treatment of osteomyelitis, time of immobilization, and the respective start of mobilization, risk analyses, and additional interventions based on the biopsychosocial model.

The overall goal of this feasibility study, as part of a quality improvement project using a CDSS, was to optimize clinical processes and, consequently, clinical outcomes [10]. Our study hypothesis was that the use of the CDSS would increase the quality of treatment, including optimal coordination, intervention, and risk prevention, and thus reduce complication rates and lengths of stay. However, the use of the CDSS did not result in a reduction in complication rates, time expenses of physicians, total cost per patient, or hospitalization time. Regarding the latter, there are many possible explanations. We had to stop the use of the CDSS at the end of the pilot phase due to the transition to another IT system that did not include the specially developed Decubitus process. This led to a relevant loss of knowledge and induced high costs. Furthermore, due to the complex health conditions of the patients and the high rate of complications, the effect of CDSS-supported care might take longer to show effects on complication rates by changing the whole culture of process management [1]. The implementation took place in a team with an established culture that had worked with paper-based structures for many years. The paper-based management was faster and more established than IT-based interprofessional solutions [10]. The slightly earlier prescription of therapies (e.g., psychology or nutritional therapy) in the CDSS group vs. the control group might be explained by the fact that during the initial acute phase of stage III/IV PI treatment, the main focus is on plastic surgery and wound care. In contrast, the Basel Decubitus Approach integrates long-term secondary prevention, such as complex risk analyses, from the beginning. These therapies are often forgotten when usual care is applied, whereas a CDSS can lead the HCPs to perform treatment in a complex way right from the beginning. In the treatment course, the members of the interprofessional team remember the necessary missing therapies, so they are then registered in the course in line with recommendations [25]. A CDSS may be more useful for inexperienced teams with restricted collaboration time when HCP turnover increases [3]. In the future, as the shortage of experienced HCPs becomes a problem for health systems, CDSS-based treatment may become more important.

The CDSS was judged as relevant and feasible by the HCPs [9], but different challenges hindered the successful integration of this project into the clinical routine. As reported in previous studies [9], IT problems, the rare use of the CDSS in the treatment of stage III/IV PI, and the increased time required for additional documentation overshadowed the positive aspects of this project [6]. Further, HCPs’ engagement with and acceptance of the CDSS decreased because the main advantage of automatic data use for different purposes was not yet possible, and analysis of big data using the CDSS data was not achieved [36].

Although some participants showed a willingness to use the CDSS, we found that misunderstandings about the purpose of the CDSS contributed to resistance by others. HCPs were disappointed with the CDSS because they interpreted its use as part of a scientific study and not as a means to improve clinical management. This underlines the importance of effective communication with users to reduce misunderstandings and resistance in a pilot study as part of a quality improvement project [37].

Although the CDSS demonstrated some advantages that could lead to future improvements in process-based clinical care, the decision regarding its implementation should be made carefully given the high investment in terms of costs and workload [3]. The commitment from different professions, the support from the executive board of the clinic, and the cultural change in the clinic to a process-based organization were helpful [3]. However, it appeared that several learning cycles were necessary to change clinical management to process management and to develop the adequate IT capacity. The knowledge gained in the development of the Basel Decubitus Approach could be transferred to other CDSS implementation in the clinical setting. Furthermore, the findings can be applied to other complex care settings.

## Study limitations

There are some limitations that should be mentioned. First, this CDSS development and implementation was the only project in the clinic that used a CDSS in an extra IT system [10, 36]. Consequently, the implementation was hindered by the infrequent use of the application [10, 36]. Integration in a broader context and different quality initiatives could have increased its impact. Second, the small sample size of the study is one of the main limitations of the analysis of this CDSS. Because a new IT system for the whole clinic was introduced soon after, the CDSS was not further developed. Third, the experiences of this CDSS project were influenced by the disappointing perception of spending money on an IT system that was subsequently abandoned. This included the complete development of this new clinic information software system. Nevertheless, the analyses of the HCPs’ perspectives and clinical parameters led to a comprehensive description of the project and can be used for further development projects.

## Conclusion

We demonstrated that the implementation of a CDSS was feasible for complex treatment procedures, such as the treatment of stage III and IV PI in people with SCI. Milestone adherence, time to prescribe, and cost capture can be used as quality indicators of the implementation. HCPs’ perspectives should always be integrated into the implementation processes, especially in clinical processes with low evidence and a need for consensus-based management. The IT processes should include reminder systems, allow multiple data use for different purposes, and provide high-speed performance to increase the acceptance by HCPs. We learned that other factors, such as the time required for training and integration with existing information systems, were also important for successful implementation and that implementation is akin to cultural development.

## Supplementary Information

**Additional file 1: Appendix Table 1.** List of use case elements and different milestones in the “Basel Decubitus Appraoch”.

**Additional file 2: Appendix Fig. 2.** CDSS overview of treatment elements (3a), of consultations (3b) and milestones in the six-week mobilisation scheme (3c). (Figures are presented in German).

**Additional file 3: Appendix Table 3.** Participants in focus groups (profession, working years in the specific profession, working years in this clinic).

**Additional file 4: Appendix Table 4.** Length of stay and profession related time invest and costs.

## Data Availability

Datasets used and analyzed during the current study are available from the corresponding author on reasonable request. All personal identifiers were removed from the dataset.

## Abbreviations

BPMN: Business Process Modeling Notation
CDSS: Clinical Decision Support System
PUAP: European Pressure Ulcer Advisory Panel
HCPs: Healthcare Professionals
ICF: International Classification of Functioning Disability and Health
PI: Pressure Injury
SCI: Spinal Cord Injury
