# Supplementary material for: Effect of a computerized decision support system on the treatment approach of stage III or IV pressure injury in patients with spinal cord injury: a feasibility study
Source: BMC Health Serv Res. 2023 Jan 31;23:103. doi: 10.1186/s12913-023-09045-y (PMC9890825; doi:10.1186/s12913-023-09045-y)
Supplement: Supplementary file 1 — Additional file 1: Appendix Table 1. List of use case elements and different milestones in the “Basel Decubitus Appraoch”. [file 12913_2023_9045_MOESM1_ESM.docx]

Effect of a computerized decision support systems in the interdisciplinary treatment of stage IV pressure injury in patients with spinal cord injury: a pragmatic pilot study

Appendix table 1: List of use case elements and different milestones in the "Basel Decubitus Appraoch"

| Management elements |  | Assessments |  | Examination |  | Consultation |
| --- | --- | --- | --- | --- | --- | --- |
| Skin and wound management |  | PI standardized documentation |  | Lung function test |  | Plastic surgeon |
| Nutrition management |  | SNST |  | Nutritional blood examination profile |  | Dietician |
| Breathing management |  | Multi morbidity index |  | Electrocardiogram |  | Physiotherapy |
| Bladder management |  | Spinal Cord independence measure III |  |  |  | Nursing |
| Bowel management |  | PI risk scale |  |  |  | Nursing |
| Spasticity |  | Modified Ashworth Scale |  |  |  |  |
| Psychological support |  | Blood pressure measurements |  |  |  | Psychology |
| Training and physical therapy in arms and legs |  | Neurological examination (ISNCSCI) |  |  |  | Physiotherapy |
| Mobility training |  | Range of motion |  |  |  | Physiotherapy |
| Auxiliary evaluation and adaptation |  |  |  |  |  | Occupational therapy |

Abbreviation: PI= pressure injury; SNST=Spinal Nutrition Screening Tool; ISNCSCI= International Standard for Neurological Classification Spinal Cord Injury
