# Supplementary material for: Effect of a computerized decision support system on the treatment approach of stage III or IV pressure injury in patients with spinal cord injury: a feasibility study
Source: BMC Health Serv Res. 2023 Jan 31;23:103. doi: 10.1186/s12913-023-09045-y (PMC9890825; doi:10.1186/s12913-023-09045-y)
Supplement: Supplementary file 2 — Additional file 2: Appendix Fig. 2. CDSS overview of treatment elements (3a), of consultations (3b) and milestones in the six-week mobilisation scheme (3c). (Figures are presented in German). [file 12913_2023_9045_MOESM2_ESM.docx]

Effect of a computerized decision support systems in the interdisciplinary treatment of stage IV pressure injury in patients with spinal cord injury: a pragmatic pilot study

Appendix figure 2: CDSS overview of treatment elements (3a), of consultations (3b) and milestones in the six-week mobilisation scheme (3c). (Figures are presented in German)


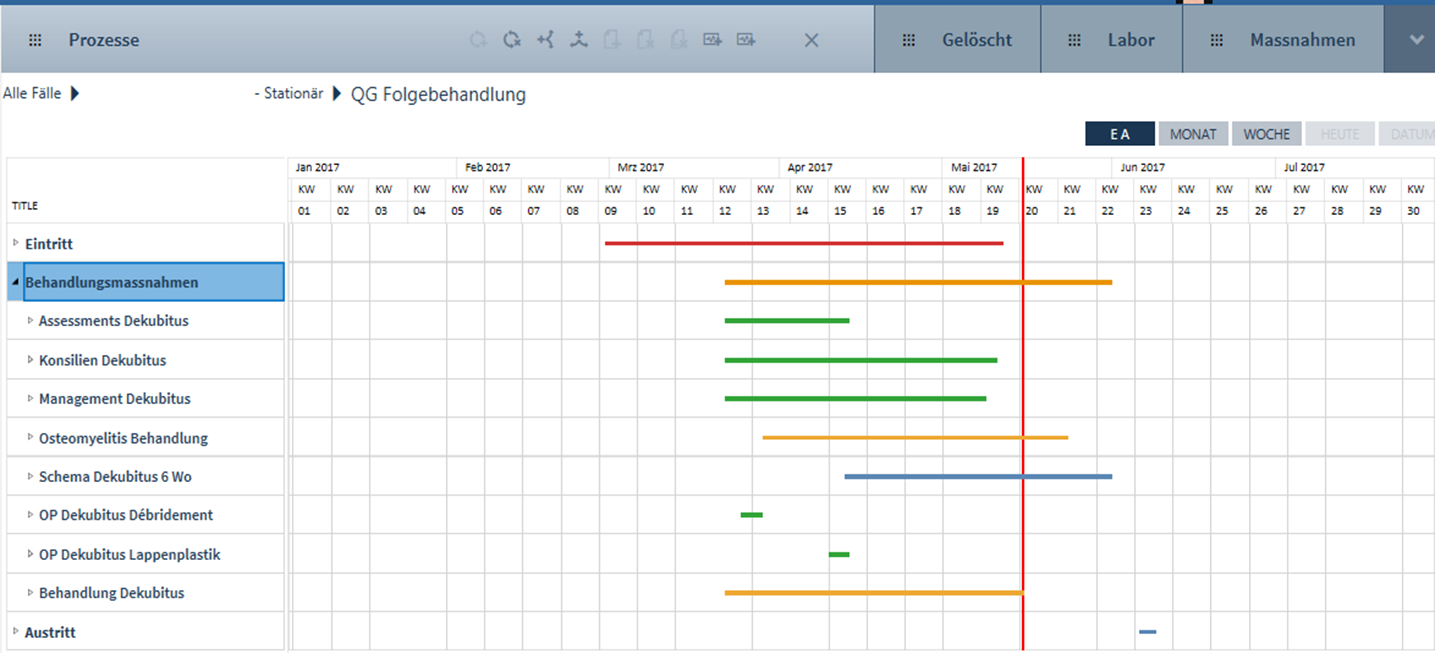


3a) Treatment elements


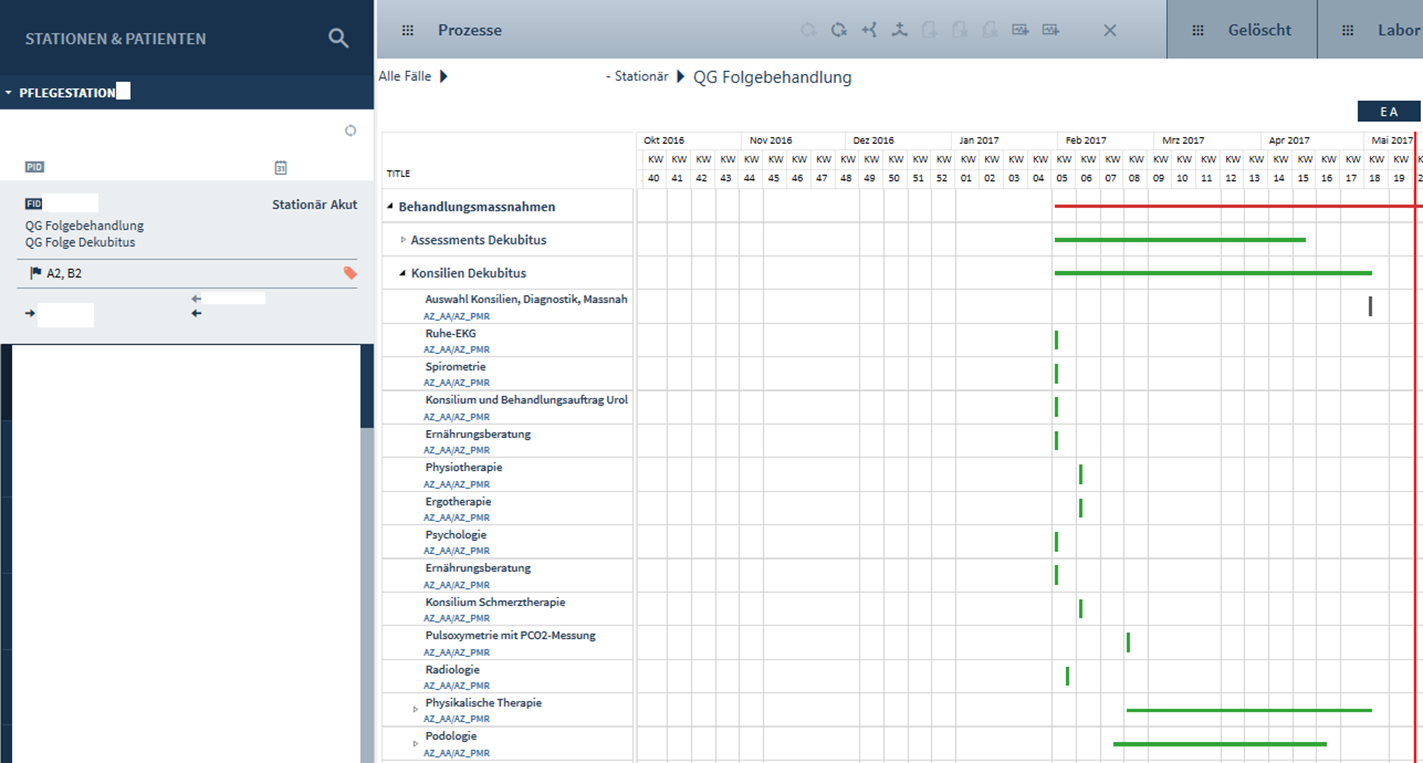


3b) consultation


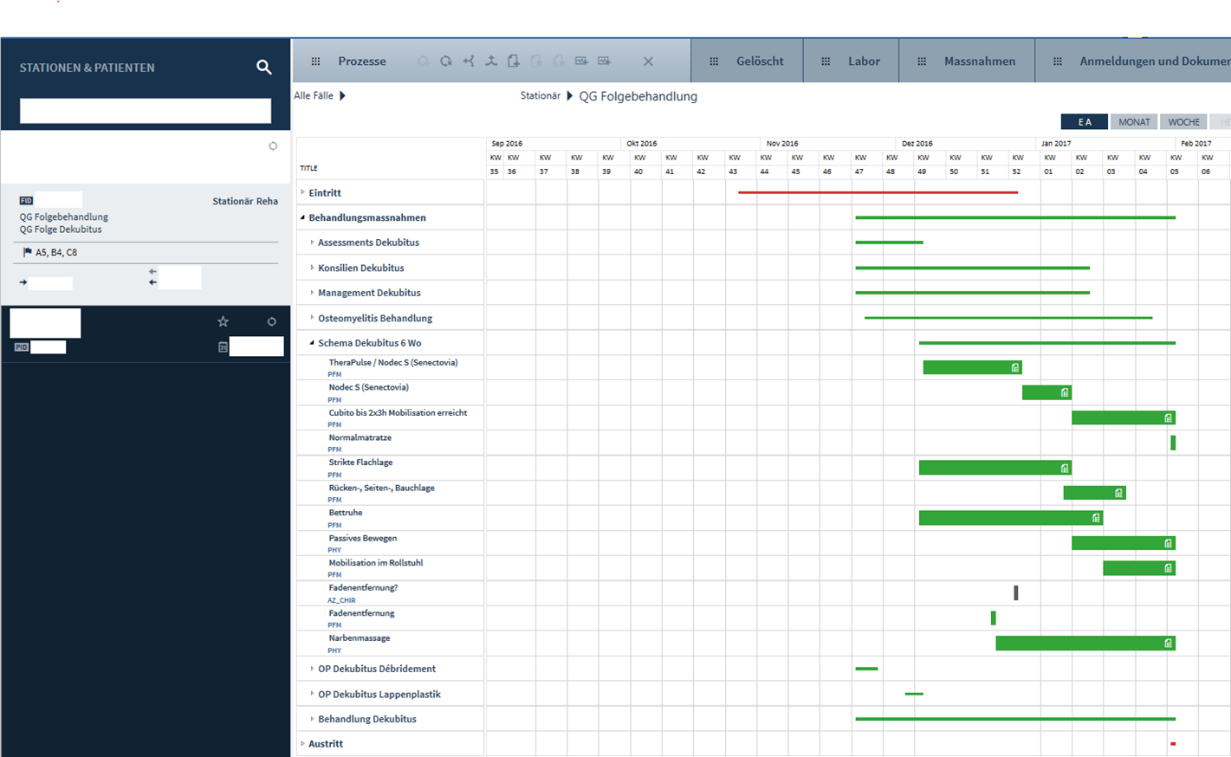


3c) milestones in the six-week mobilisation scheme
