# Supplementary material for: Effect of a computerized decision support system on the treatment approach of stage III or IV pressure injury in patients with spinal cord injury: a feasibility study
Source: BMC Health Serv Res. 2023 Jan 31;23:103. doi: 10.1186/s12913-023-09045-y (PMC9890825; doi:10.1186/s12913-023-09045-y)
Supplement: Supplementary file 3 — Additional file 3: Appendix Table 3. Participants in focus groups (profession, working years in the specific profession, working years in this clinic). [file 12913_2023_9045_MOESM3_ESM.docx]

Effect of a computerized decision support systems in the interdisciplinary treatment of stage IV pressure injury in patients with spinal cord injury: a pragmatic pilot study

Appendix table 3: Participants in focus groups (profession, working years in the specific profession, working years in this clinic)

| Participant characteristics (sample n=30) | | n (%) |
| --- | --- | --- |
| Sex | |  |
|  | Female | 26 (87%) |
|  | Male | 4 (13%) |
| Profession | |  |
|  | Nurse | 8 (27%) |
|  | Physical therapist | 6 (20%) |
|  | Occupational therapist | 3 (10%) |
|  | Physician | 13 (43%) |
| Years in profession | |  |
|  | <5 years | 8 (27%) |
|  | 5-10 years | 6 (20%) |
|  | 11-20 years | 7 (23%) |
|  | 21-30 years | 7 (23%) |
|  | 31-40 years | 2 (7%) |
| Years in this clinic | |  |
|  | <5 years | 13 (43%) |
|  | 5-10 years | 4 (13%) |
|  | 11-20 years | 11 (37%) |
|  | 21-30 years | 2 (7%) |

Abbreviation: n= number
