# Supplementary material for: Effect of a computerized decision support system on the treatment approach of stage III or IV pressure injury in patients with spinal cord injury: a feasibility study
Source: BMC Health Serv Res. 2023 Jan 31;23:103. doi: 10.1186/s12913-023-09045-y (PMC9890825; doi:10.1186/s12913-023-09045-y)
Supplement: Supplementary file 4 — Additional file 4: Appendix Table 4. Length of stay and profession related time invest and costs. [file 12913_2023_9045_MOESM4_ESM.docx]

Effect of a computerized decision support systems in the interdisciplinary treatment of stage IV pressure injury in patients with spinal cord injury: a pragmatic pilot study

Appendix table 4: Length of stay and profession related time invest and costs

| **Parameter** | **Statistic** | **Total**  **(N=30)** | **CDSS Group**  **(N=15)** | **Control Group**  **(N=15)** | **P-value** |
| --- | --- | --- | --- | --- | --- |
| Length of Stay (days) | Mean (SD) | 90 (26.6) | 98 (28.3) | 81 (22.8) | 0.124 |
|  | Median (Q1; Q3) | 80 (71; 117) | 89 (76; 120) | 78 (71; 86) |  |
|  | Min; Max | 54; 147 | 55; 147 | 54; 140 |  |
| Time per Week (hours) | | |  |  |  |
| DP | Mean (SD) | 62 (15.5) | 65 (16.5) | 59 (14.3) | 0.245 |
|  | Median (Q1; Q3) | 57 (53; 73) | 70 (53; 74) | 56 (53; 60) |  |
|  | Min; Max | 31; 104 | 31; 95 | 41; 104 |  |
| IP | Mean (SD) | 35 (17.2) | 33 (17.5) | 37 (17.2) | 0.604 |
|  | Median (Q1; Q3) | 32 (22; 49) | 32 (21; 43) | 29 (22; 54) |  |
|  | Min; Max | 12; 78 | 12; 78 | 15; 65 |  |
| Total | Mean (SD) | 98 (28.6) | 99 (29.5) | 97 (28.7) | 0.820 |
|  | Median (Q1; Q3) | 97 (77; 115) | 104 (69; 110) | 82 (77; 118) |  |
|  | Min; Max | 43; 169 | 43; 161 | 62; 169 |  |
| Total Costs | Mean (SD) | 142.7 (57.6) | 164.6 (59.8) | 120.9 (47.6) | 0.049 |
| (in 1,000 CHF) | Median (Q1; Q3) | 136.5 (103.6; 189.8) | 159.0 (110.3; 204.6) | 120.7 (95.1; 151.9) |  |
|  | Min; Max | 22.6; 277.2 | 78.4; 277.2 | 22.6; 191.0 |  |
| Costs per Profession (in 1,000 CHF) | | |  |  |  |
| Medical | Mean (SD) | 12.3 (4.9) | 13.2 (4.6) | 10.3 (5.5) | 0.254 |
|  | Median (Q1; Q3) | 11.9 (9.2; 16.6) | 13.1 (10.4; 17.1) | 10.3 (6.7; 15.7) |  |
|  | Min; Max | 1.9; 19.6 | 3.7; 19.6 | 1.9; 16.6 |  |
| Therapeutic | Mean (SD) | 12.5 (7.2) | 15.0 (8.4) | 10.0 (4.9) | 0.071 |
|  | Median (Q1; Q3) | 9.9 (8.7; 15.3) | 11.0 (9.2; 20.1) | 9.1 (7.4; 10.5) |  |
|  | Min; Max | 1.7; 35.9 | 6.0; 35.9 | 1.7; 22.6 |  |
| Plastic Surgery | Mean (SD) | 11.3 (8.4) | 13.3 (10.7) | 9.3 (4.7) | 0.520 |
|  | Median (Q1; Q3) | 8.8 (5.9; 15.2) | 9.2 (5.8; 15.4) | 8.5 (6.5; 10.4) |  |
|  | Min; Max | 2.1; 43.5 | 4.0; 43.5 | 2.1; 17.6 |  |
| Nursing | Mean (SD) | 58.4 (25.8) | 67.7 (27.9) | 49.0 (20.4) | 0.071 |
|  | Median (Q1; Q3) | 52.0 (41.7; 71.0) | 65.7 (46.8; 85.0) | 48.4 (34.9; 69.3) |  |
|  | Min; Max | 8.2; 121.8 | 29.4; 121.8 | 8.2; 80.8 |  |

Abbreviation: SD= standard deviation; Q= Quartile; DP= daily practice; IP= interprofessional coordination; CDSS = Computerized Decision Support System
